# Atavistic Genetic Expression Dissociation (AGED) During Aging: Meta‐Phylostratigraphic Evidence of Cellular and Tissue‐Level Phylogenetic Dissociation

**DOI:** 10.1111/acel.70305

**Published:** 2025-12-08

**Authors:** Léo Pio‐Lopez, Michael Levin

**Affiliations:** ^1^ Allen Discovery Center Tufts University Medford Massachusetts USA; ^2^ Wyss Institute for Biologically Inspired Engineering Harvard University Boston Massachusetts USA

**Keywords:** aging, longevity, phylostratigraphy

## Abstract

Aging is commonly attributed to accumulated damage, or evolved antagonistic genetic trade‐offs, which lead to an accumulation of damage causing misexpression of genes necessary for longevity. We propose an atavistic dysregulation of gene expression at cellular and tissue levels during aging, framing aging as a gradual regression toward ancestral cellular states. Similarly to the atavistic model of cancer, in which cells revert to unicellular‐like behavior, aging may result from the breakdown of coordinated morphogenetic control, leading organs and tissues toward less integrated, ancient unicellular states. We suggest that aging may involve a progressive reversal of the well‐known ontogenetic tracing of prior phylogenetic embryonic characteristics. Moreover, aging could involve a loss of large‐scale coordination, with tissues reverting to ancient gene expression to different degrees. We tested this hypothesis using a meta‐phylostratigraphic analysis, finding: (1) An atavistic over‐representation of differential expression in the most ancient genes and under‐representation in the evolutionary youngest genes for two multi‐tissue aging databases, and tissues covering skin, ovarian, immune, senescent and mesenchymal‐senescent cells; (2) No significant atavistic over‐representation of the differential gene expression during aging of brain cells and mesenchymal stem cells; (3) overall age‐dependent increase of heterogeneity in the direction of the phylogenetic position of tissues' transcriptional profiles; (4) and an overall negative evolutionary age mean shift toward the most ancient genes. Our analyses suggest that aging involves uncoordinated and tissue‐specific phylogenetic changes in gene expression. Understanding aging as a structured, heterogeneous atavistic process opens new avenues for rejuvenation, focusing on restoring multicellular coherence in evolutionarily youthful gene expression.

## Graphical abstract

Aging represents an atavistic over‐representation of differential expression in the most ancient genes and under‐representation in the evolutionary youngest genes for two multi‐tissue aging databases, and tissues covering skin, ovarian, immune, senescent and mesenchymal‐senescent cells. However, some tissues like brain and mesenchymal stem cells do not show significant atavistic shifts. Overall, aging reflects a tissue‐specific, uncoordinated genetic atavistic dissociation, opening new perspectives for rejuvenation targeting multicellular integration and youthful gene expression patterns.

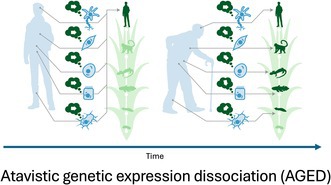

## 1 Introduction

### 1.1 Aging Theories

Most complex organisms experience a sequential life course consisting of embryonic development stages, growth to maturity, and then functional decline and ultimately death. Aging leads to progressive deterioration of cellular structures and biological functions in living systems (de Magalhães 2011, 2023; Gladyshev et al. 2021; Austad and Hoffman 2018). The aging process is influenced by diverse elements including genetic inheritance, environmental conditions, and behavioral choices (Partridge et al. 2018). The resistance of biological systems to noise and damage decreases with age (Rubin 2007, 2006; Rubin et al. 1996), leading to health issues including cardiovascular complications, cancer, neurodegenerative disorders, metabolic disruptions such as type II diabetes, and compromised immune responses to pathogens, which strongly impact quality of life (Gladyshev et al. 2021; Partridge et al. 2018; López‐Otín et al. 2023; Hayflick 2007).

The scientific community has been focused on two main theoretical frameworks to understand the aging process: damage‐based (Gladyshev et al. 2021; López‐Otín et al. 2023) and programmatic (de Magalhães 2011, 2023; Gladyshev et al. 2021; Kirkwood and Melov 2011; Lidsky et al. 2023, 2022; Lidsky and Andino 2022, 2020) theories. Damage‐based theories hold that aging results from the inevitable accumulation of molecular damage over time. Essential cellular components including genetic stability, telomere length, mitochondrial activity, and protein homeostasis experience severe degradation. In contrast, programmatic theories propose that aging is the evolutionary result of a genetically encoded biological trajectory. This perspective suggests that the aging process is encoded in our genome, because it provides a reproductive advantage at the lineage level, rather than merely representing stochastic damage accumulation over time (de Magalhães and Church 2005; Gems 2022; Skulachev and Skulachev 2014).

### 1.2 Longevity as the Result of Anatomical Homeostasis

A novel direction with respect to aging is suggested by a focus on morphogenesis and morphostasis (cellular self‐assembly into complex anatomical forms, and continued maintenance of the correct structure) as active navigation of anatomical morphospace (Fields and Levin 2022; Harris 2018; Pezzulo and Levin 2016, 2015; Rasskin‐Gutman and Izpisua‐Belmonte 2004; Stone 1997). This framework sees embryogenesis, regeneration, and cancer suppression as a continuous dynamic set of decisions made by the cellular collective to reach and maintain organ‐level target morphologies (Fields and Levin 2022; Pezzulo and Levin 2015; Levin 2023a). Numerous tools from behavioral neuroscience and cybernetics have been deployed in morphogenetic systems to probe the mechanisms that coordinate cells toward common anatomical endpoints (Pezzulo and Levin 2016, 2015; Levin 2023b; Friston et al. 2015). The ability to reach and maintain species‐specific large‐scale anatomical states reliably, despite noise and perturbations, is a kind of homeostatic dynamic that has been modeled as a collective intelligence (McMillen and Levin 2024), with many applications in biomedicine across birth defects and regeneration (Lagasse and Levin 2023; Levin 2024). Moreover, this approach has provided novel ways to address cancer as a loss of coordination of cells toward tissue‐level homeostatic goals (Levin 2021; Moore et al. 2017; Chernet and Levin 2013). From this perspective, one can ask what kind of information‐processing disorders could contribute to aging as a long‐term loss of the ability to organize cells and subcellular materials toward maintenance of a healthy complex form.

We recently proposed that aging is the result of the loss of morphostatic information (Pio‐Lopez and Levin 2024; Pio‐Lopez et al. 2024). In this framework, aging is an emergent failure mode of information‐processing collectives that lose morphogenetic guidance after the construction of the adult body structure has been completed (Pio‐Lopez and Levin 2024; Pio‐Lopez et al. 2024). What kind of directional changes might be observed in cells and tissues after the primary anatomical goals have been met, in addition to loss of precision and random alterations? Moreover, when not bound to a single (body‐wide) anatomical setpoint as occurs during embryogenesis, would different cells in the body eventually adopt distinct setpoints, thus resulting in the loss of coherent function as observed in aging?

The setpoints for anatomical homeostasis in evolved creatures are shaped by evolution (Newman and Bhat 2009; McGhee 2007; Ollé‐Vila et al. 2016; Duran‐Nebreda et al. 2016; Raff 1996; Gilbert et al. 1996; Jeffery and Raff 1983); specifically, they are determined by phylogenetic position and the body plan it specifies. Could the kind of dissociation of self‐model from reality that is observed in certain psychological disease states (Nijenhuis et al. 1998; Waller et al. 2001) have a somatic counterpart, in which the actual phylogenetic age of cells ( *Homo sapiens* ) becomes dissociated from the *effective* physiological phylogenetic age due to the expression of more ancient genes? We sought to ask: what is the relationship between an organism's age and the phylogenetic information its tissues attempt to implement? Using transcriptional profiling datasets, and phylostratigraphy (Domazet‐Lošo et al. 2007; Domazet‐Lošo and Tautz 2010; Lineweaver and Davies 2021) to analyze the evolutionary age of genes being expressed in specific tissues, we tested two hypotheses. First, that aging involves a roll‐back of transcriptional profiles to include more ancient genes (in effect, a reversal of the well‐known “ontogeny recapitulates phylogeny” dynamic with respect to embryonic patterns). Second, that this would include a significant loss of *coordination* across the body, in which different tissues ended up phenocopying the transcriptional states of different positions across phylogenetic history, similarly to what occurs in cancer (Levin 2021; Bussey et al. 2017; Davies and Lineweaver 2011; Rubin 1985).

### 1.3 The Atavistic Disregulation Hypothesis of Aging

We tested this hypothesis using a meta‐phylostratigraphic analysis. We applied a phylostratigraphic analysis using RNA‐seq and scRNA‐seq data from 8 different studies and two meta‐analyses including different tissues RNA‐seq during aging (Palmer et al. 2021; Tikhonov et al. 2024), scRNA‐seq data from skin cells (Zou et al. 2021), an aging meta‐analysis of the scRNA‐seq of senescent cells (Avelar et al. 2020), and brain (cortex, hippocampus and cerebellum cells) (González‐Velasco et al. 2020), immune (Lu et al. 2022), ovarian (Jin et al. 2024) and stem cells RNA‐seq (Wagner et al. 2009) (see Table 1). We found: (1) An atavistic over‐representation of differential expression in the most ancient genes and under‐representation in the evolutionary youngest genes for two multi‐tissue aging databases, and tissues covering skin, ovarian, immune, senescent and mesenchymal‐senescent cells; (2) No significant atavistic over‐representation of the differential gene expression during aging of brain cells and mesenchymal stem cells; (3) overall age‐dependent increase of heterogeneity in the direction of the phylogenetic position of tissues' transcriptional profiles; and (4) an overall negative evolutionary age mean shift toward the most ancient genes. Thus, we propose that while cancer is a loss of cellular organization in space, aging involves a loss of cellular organization in (evolutionary) time.

**TABLE 1:** Mann–Whitney *U* test results for combined gene sets across multiple aging datasets.

Dataset	Mann–Whitney *p*‐value	Significance	Mean age shift
GenAge	**1.052e‐36**	***	**−1.63**
AgeMeta	**1.134e‐12**	***	**−1.73**
Skin_40‐69	**1.577e‐18**	***	**−2.23**
Skin_70+	**1.577e‐03**	**	**−0.77**
Ovary	**3.098e‐59**	***	**−1.96**
Progenitors	**3.106e‐20**	***	**−1.84**
Mesenchymal_senescent	**2.276e‐43**	***	**−2.41**
CellAge_Senescence	**3.684e‐40**	***	**−2.32**
Brain_Cortex	**3.629e‐04**	***	**−0.74**
Brain_Hippocampus	9.120e‐02	ns	**−**0.31
Brain_Cerebellum	**4.469e‐04**	***	**−0.9**
Mesenchymal	5.313e‐01	ns	**−**0.27
CD8T	**7.218e‐11**	***	**−1.47**

*Note:* *p*‐values indicate the significance of distribution differences between gene sets and the baseline evolutionary age distribution. Mean age shift represents the average change in evolutionary age categories, where negative values indicate a shift toward older evolutionary ages. Significance levels: ***p < 0.001, **p < 0.01. Bold text indicates significant results (*p* < 0.05).

## 2 Material and Methods

We applied a meta‐phylostratigraphic analysis using RNA‐seq and scRNA‐seq data from 8 different studies two meta‐analyses including different tissues: RNA‐seq during aging (Palmer et al. 2021; Tikhonov et al. 2024), scRNA‐seq data from skin cells (Zou et al. 2021), an aging meta‐analysis of the scRNA‐seq of senescent cells (Avelar et al. 2020), and brain (cortex, hippocampus and cerebellum cells) (González‐Velasco et al. 2020), immune (Lu et al. 2022), ovarian (Jin et al. 2024) and stem cells RNA‐seq (Wagner et al. 2009) (see Table 2).

**TABLE 2:** Table of all datasets used in this study for the phylostratigraphic meta‐analysis of aging genetic expression.

Cell types	All tissues, meta‐analysis	Skin cells		Senescent cells	Brain cells			Immune cells	Ovarian cells	Stem cells		
Data	Age‐related genes	scRNA‐seq, 40–70 years old	scRNA‐seq, > 70 years old	Meta‐analysis	Brain cortex	Hippocampus	Cerebellum	CD8T	Ovary	MSC	MSC‐senescent	HPC
References	Palmer et al. (2021); Tikhonov et al. (2024)	Zou et al. (2021)		Avelar et al. (2020)	González‐Velasco et al. (2020)			Lu et al. (2022)	Jin et al. (2024)	Wagner et al. (2009)		

*Note:* Eight datasets were used; several of them are meta‐analyses totaling more than 100 datasets.

To analyze the differentially expressed genes (DEGs) in the aging cells under various conditions through phylostratigraphic analysis, we used the evolutionary ages of 19,660 human protein‐coding genes as determined by Litman and Stein (2019). These genes were categorized into 19 major phylostrata, as outlined by Domazet‐Lošo and Tautz (2010). The phylostrata are a hierarchical range of evolutionary origins for: all living organisms (including Eubacteria, Bacteria and their descendants, e.g., unicellulars), Eukaryota, Opisthokonta, Holozoa, Metazoa, Eumetazoa, Bilateria, Deuterostomia, Chordata, Olfactores, Craniata, Euteleostomi, Tetrapoda, Amniota, Mammalia, Boreoeutheria, Eutheria, Euarchontoglires, and Primates.

We quantified the number of genes expressed under the various experimental conditions to assess the atavistic patterns we may find during aging in terms of genetic expression. We then applied an overrepresentation test for each group of genes in each phylostrata compared to the ensemble of ages of all human protein‐coding genes. The overrepresentation test (also known as enrichment analysis) is a widely used statistical method to determine whether a specific set of elements (e.g., genes, proteins, or other features) is significantly over‐represented in a given category compared to a background distribution. Over‐representation tests are extensively used in various scientific fields, particularly in Gene Ontology (GO) Enrichment Analysis where we identify functional categories over‐represented in a set of differentially expressed genes (Ashburner et al. 2000), Pathway Enrichment Analysis where we assess whether biological pathways, such as KEGG pathways, are significantly enriched in a dataset (Kanehisa et al. 2017) and Disease Association Studies where we link genetic variants to diseases by testing for enrichment in disease‐associated categories (Subramanian et al. 2005). This approach is crucial for understanding biological processes, molecular pathways, and functional annotations associated with high‐throughput data.

More specifically, in our case, given a “gene universe” (here the list of all human protein‐coding genes) containing *N* total genes, this test evaluates whether a specific phylostratigraphic category is statistically overrepresented within a smaller subset of *n* genes selected from this gene universe in a specific condition. With *M* genes belonging to a specific phylostratigraphic category in the gene universe, the test calculates the probability of observing *k* genes from this functional category within the selected subset of *n* genes randomly, using the hypergeometric distribution:

$$P\left(X=K\right)=\frac{\left(\begin{array}{c} M\\ k \end{array}\right)\left(\begin{array}{l} N-M\\ n-k \end{array}\right)}{\left(\begin{array}{l} N\\ n \end{array}\right)}$$

A *p*‐value is then calculated to determine the significance of the over‐representation (*X* ≥ *k*) and under‐representation (*X* ≤ *k*) of the observed enrichment. Here we use a *p*‐value inferior to 0.05 to determine the significance, with Benjamini–Hochberg False Discovery Rate (FDR) correction applied to control for multiple testing across all 19 evolutionary age strata within each dataset. We also performed a Mann–Whitney test to assess mean evolutionary age shifts in the overall distribution of evolutionary ages compared to the baseline distribution.

## 3 Results

### 3.1 Atavistic Over‐Representation in the Most Ancient Genes for Multi‐Tissues Aging Signatures (GenAge and AgeMeta) Skin, Ovarian, Immune, Senescent, and Mesenchymal‐Senescent Cells

**FIGURE 1:**
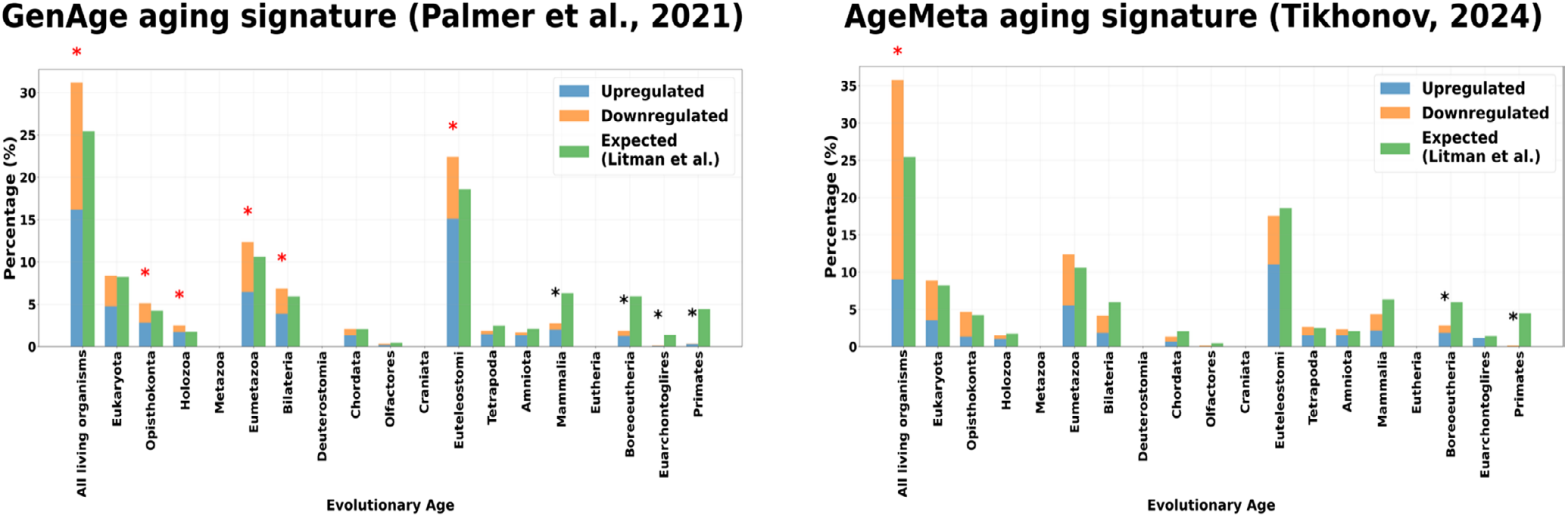
Atavistic over‐representation observed in the GenAge and AgeMeta aging signatures. The figures illustrate gene expression changes in different cell types (skin, immune, and ovarian cells) across evolutionary age categories. The bar charts separate genes into upregulated (blue) and downregulated (orange) categories compared to the expected distribution (green), providing insight into how aging influences gene regulation across different phylogenetic layers. The star above a bar chart means that the over‐representation test is significant (red for over‐representation test, black for under‐representation test, * = *p* < 0.05 with FDR‐correction).

We applied the phylostratigraphic analysis on two multi‐tissue aging datasets. The first signature was extracted from GenAge (Palmer et al. 2021) and has been extracted from 127 publicly available microarray and RNA‐Seq datasets from mice, rats and humans, identifying a transcriptomic signature of aging across species and tissues (brain, heart and muscle). The second one, AgeMeta, is likewise a meta‐analysis of 51 humans scRNA‐seq datasets of different tissues (vastus lateralis, cerebellum, frontal cortex, muscle, brain, adipose tissue, adrenal gland, blood, blood vessel, brain (without cerebellum), esophagus, heart, lung, nerve, pituitary, salivary gland, prostate, testis, and thyroid). We observed in both aging signatures an over‐representation of the most ancient genes in the “All living organisms” strata (see Figure 1). The GenAge aging signature shows more over‐representations, notably in Ospithokonta, Holozoa, Eumetazoa, Bilateria and Euteleostomi strata while the human AgeMeta signature shows only one over‐representation in the unicellular genes. The number of upregulated and downregulated genes in the most ancient genes in the GenAge aging signature is almost equal, suggesting more a reshuffling in the genetic expression during aging in the most ancient genes. The AgeMeta aging signature is dominated by downregulated genes in the “All living organisms” strata.

Interestingly, we also found an under‐representation in the evolutionary youngest genes for the GenAge signature for Mammalia, Boroeutheria, Euarchontoglires, and Primates. Similarly, we also observed an important under‐representation for the AgeMeta signature in Boroeutheria and Primates strata. Overall, the most important changes were noted in the most ancient genes, while we observed an under‐representation of the changes in the youngest ones compared to the expected distribution.

Both meta‐signatures showed significant shifts toward evolutionarily older genes, with mean age shifts of −2.41 and −2.32 categories respectively (see Table 1).

We conclude that there is an atavistic over‐representation of differentially expressed genes in both aging signatures with a heterogeneity in the direction of the genetic changes.

#### 3.1.1 Atavistic Over‐Representation During Aging in Skin, Ovarian, Immune, Senescent, and Mesenchymal‐Senescent Cells

**FIGURE 2:**
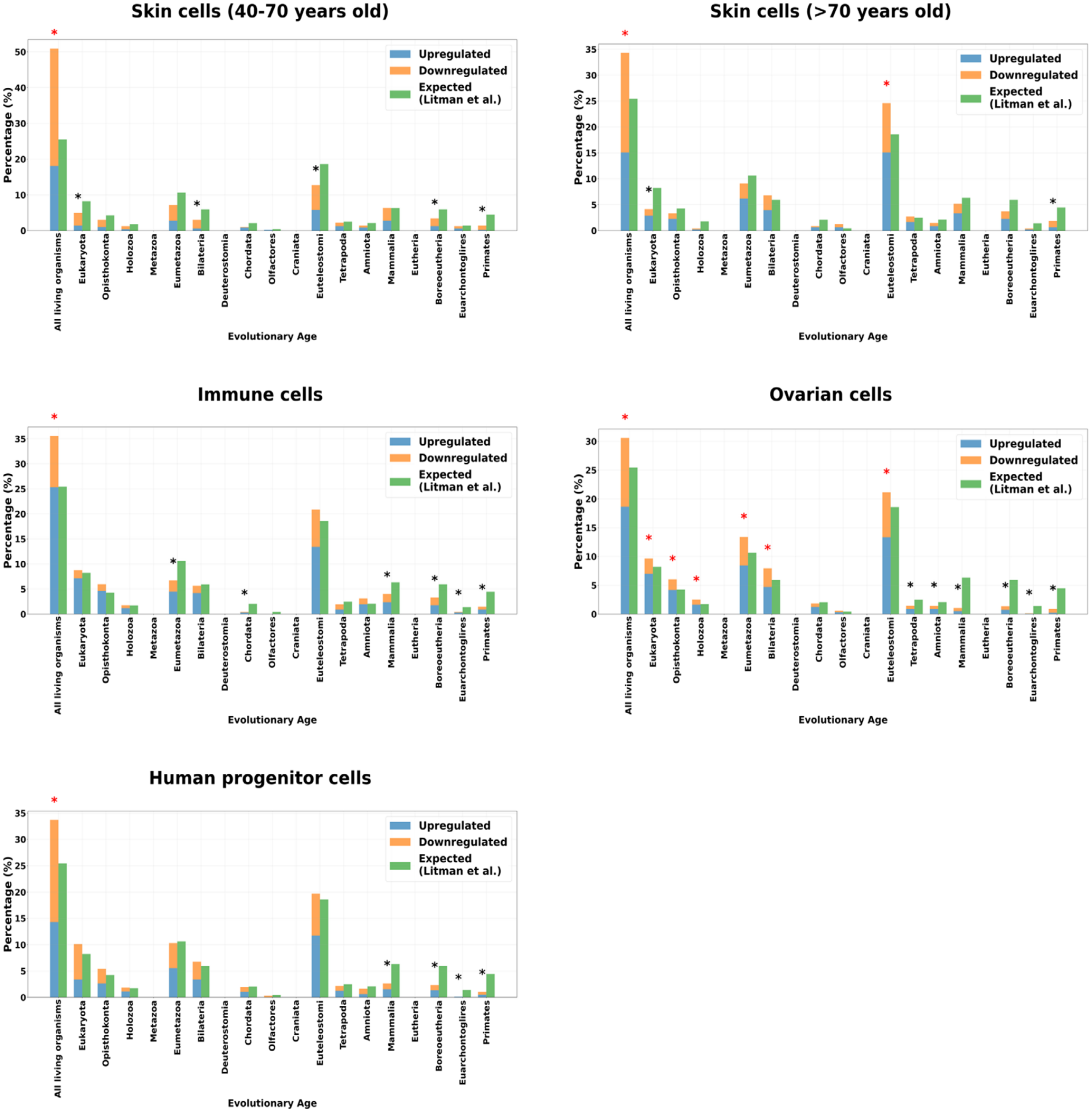
Atavistic over‐representation observed in the skin, immune and ovarian cells. The figures illustrate gene expression changes in different cell types (skin, immune, and ovarian cells) across evolutionary age categories. The bar charts separate genes into upregulated (blue) and downregulated (orange) categories compared to the expected distribution (green), providing insight into how aging influences gene regulation across different phylogenetic layers. The star above a bar chart means that the over‐representation test is significant (red for over‐representation test, black for under‐representation test, * = *p* < 0.05 with FDR‐correction).

We applied the phylostratographic analysis to skin, ovarian and immune scRNA‐seq. For skin cells, in both age groups, gene expression changes are most significant in the most ancient evolutionary category, for example, “All living organisms”. A shift is observed in gene percentages across aging, with a decrease in downregulated genes in the oldest group. In the 40–70 years age group, genes belonging to “All living organisms” show the highest number of expression changes, with a stronger contribution from downregulated genes. In individuals older than 70, the “All living organisms” and “Euteleostomi” categories showed a significant increase in gene expression changes, particularly upregulation for the latter. A notable decrease in gene expression changes is seen in “All living organisms” in the > 70 group compared to the 40–70 group of fibroblasts with < 35% of the changes in this stratum (see Figure 2). The under‐representation is more widespread for the skin cells. In the 40–70 group of fibroblasts, we have a significant under‐representation for “Eukaryota”, “Bilateria”, “Euteleostomi”, “Boroeutheria” and “Primates”. We have an under‐representation for the > 70 group only for “Eukaryota” and “Primates”.

As in skin cells, the highest number of over‐represented differentially expressed genes in immune cells was found in “All living organisms”. Compared to skin cells 40–70 group, immune cells exhibited fewer gene expression changes in the most ancient genes. Upregulated genes were more important in the most ancient genes (see Figure 2). We found an under‐representation for “Eumetazoa”, “Chordata”, “Mammalia”, “Boroeutheria”, “Euarchontoglires”, and “Primates”.

Ovarian cells showed the highest total number of differentially expressed genes among all cell types. The most significant gene expression changes occurred in ancient evolutionary categories. From “All living organisms” to “Bilateria” strata, we observed an over‐representation of the genetic changes (see Figure 2). Ovarian cells also showed a higher level of gene expression shifts (−1.96, see Table 1), possibly reflecting the pronounced effects of aging in reproductive tissues. The under‐representation is clearly located in the youngest genes, from “Tetrapoda” to “Primates” strata.

Human progenitor cells showed a significant atavistic over‐representation in the “All living organisms” evolutionary age (see Figure 2). We observed mostly down‐regulation in the genetic expression in this over‐representation. As in ovarian cells, the under‐representation is located in the evolutionary youngest genes, from “Mammalia” and “Primates”.

**FIGURE 3:**
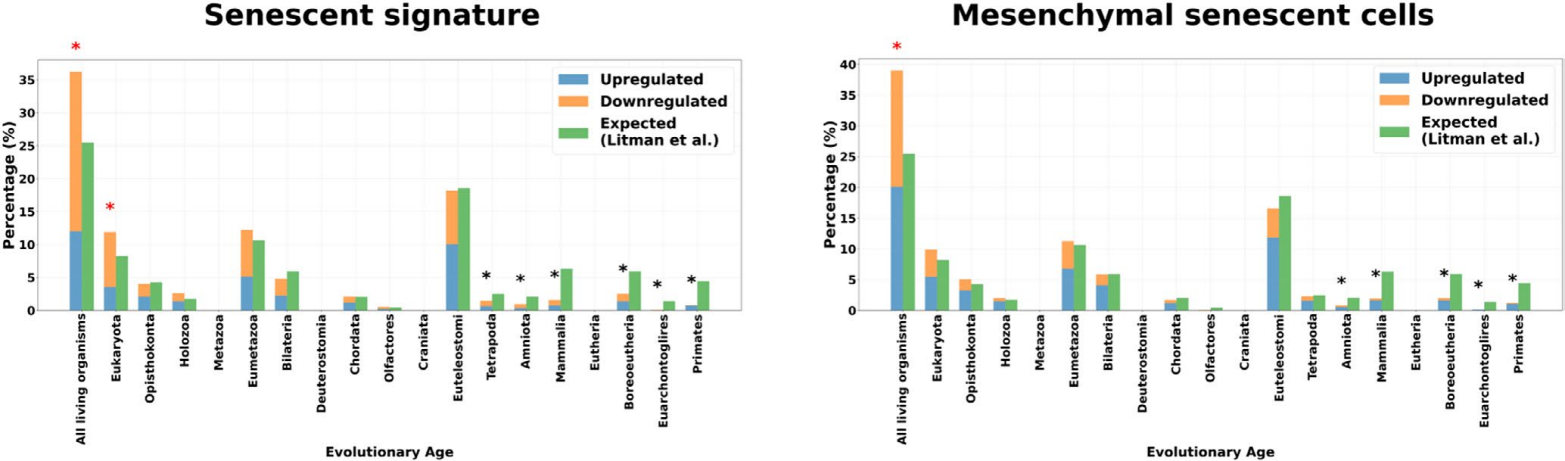
Atavistic over‐representation found in the senescent genetic signature and mesenchymal‐senescent cells. The figures illustrate gene expression changes in different cell types (skin, immune, and ovarian cells) across evolutionary age categories. The bar charts separate genes into upregulated (blue) and downregulated (orange) categories compared to the expected distribution (green), providing insight into how aging influences gene regulation across different phylogenetic layers. The star above a bar chart means that the over‐representation test is significant (red for over‐representation test, black for under‐representation test, * = *p* < 0.05 (FDR‐corrected]).

In the CellAge senescent signature, gene expression changes are most pronounced in ancient evolutionary categories, with “All living organisms” showing the highest level of differentially expressed genes, dominated by downregulation. The stratum “Eukaryota” also exhibits significant over‐representation (see Figure 3). The under‐representation is also largely located in the youngest genes, from “Tetrapoda” and “Primates” strata.

Mesenchymal senescent cells followed a similar trend, with “All living organisms” stratum showing significant over‐representation, though the magnitude is slightly higher than in the general senescent signature (see Figure 3). We found an under‐representation mainly located in the youngest genes, from “Amniota” to “Primates”.

Tissue‐specific aging signatures exhibited significant negative evolutionary age shifts: skin cells 40–70 (−2.23), ovary (−1.96), and progenitor cells (−1.84) showed strong shifts toward older genes, while CD8+ T‐cells (−1.47) and skin > 70 (−0.77) displayed a more moderate atavistic pattern, indicating tissue‐specific manifestations of evolutionary regression during aging (see Table 2). Senescence models (Mesenchymal senescent, CellAge senescence) gene sets demonstrated consistent shifts toward evolutionarily older genes (−2.41 and −2.32 categories).

Overall, we conclude that there is an atavistic overrepresentation in the differential genetic expression during aging in skin, immune, ovarian and human progenitor cells associated with a heterogeneity in the direction of the changes; sometimes the atavistic change is more about downregulation (skin cells), sometimes the opposite with more upregulation in the most ancient genes (immune and ovarian cells).

#### 3.1.2 No Atavistic Genetic Over‐Representation During Aging in Brain Cells and Mesenchymal Stem Cells

**FIGURE 4:**
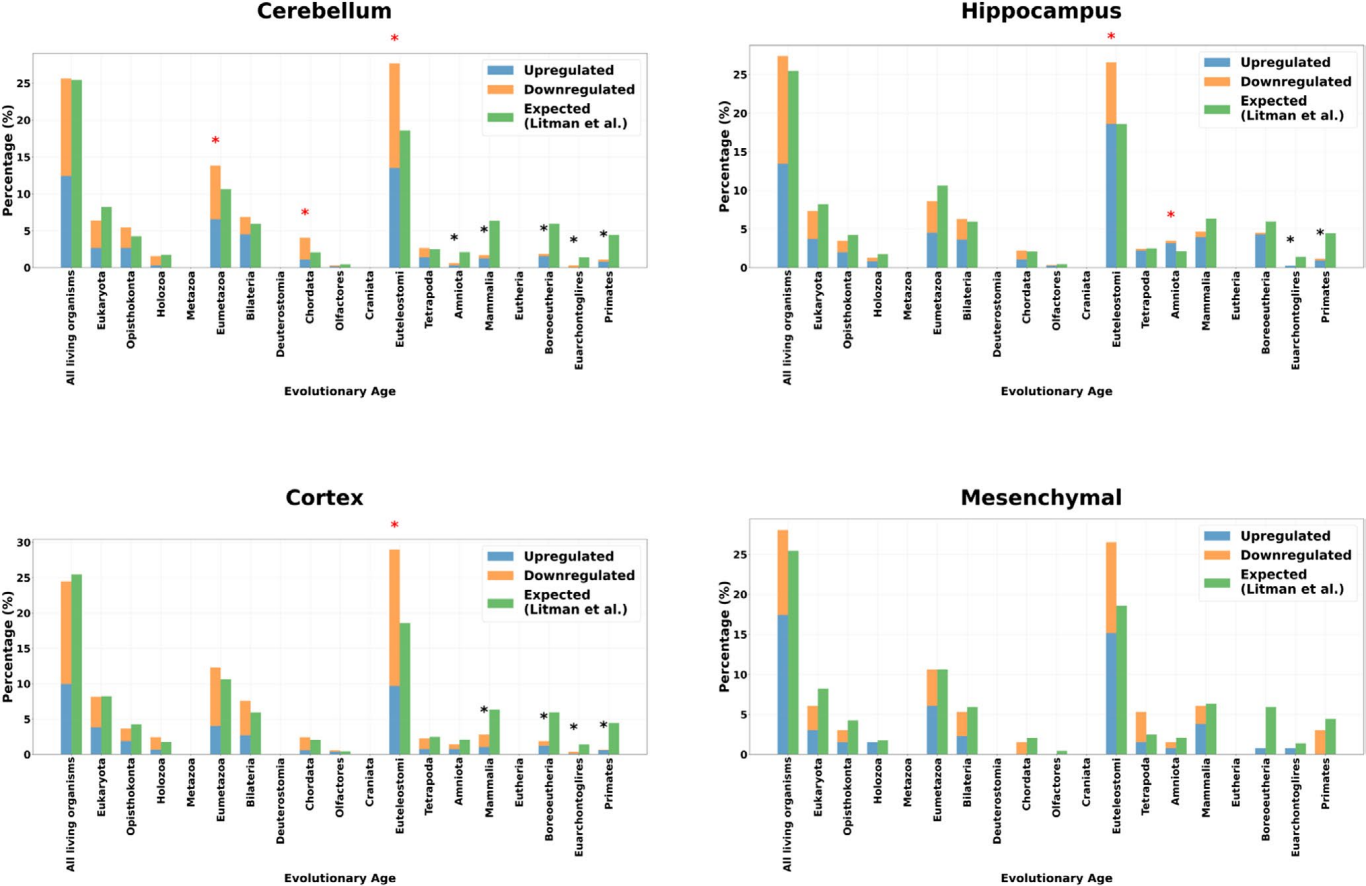
No atavistic genetic over‐representation is found during aging in brain cells and mesenchymal stem cells. The figures illustrate gene expression changes in different cell types (skin, immune, and ovarian cells) across evolutionary age categories. The bar charts separate genes into upregulated (blue) and downregulated (orange) categories compared to the expected distribution (green), providing insight into how aging influences gene regulation across different phylogenetic layers. The star above a bar chart means that the over‐representation test is significant (red for over‐representation test, black for under‐representation test, * = *p* < 0.05 with FDR‐correction).

Cerebellum, hippocampus and cortex cells exhibited similar patterns, with an over‐representation of differentially expressed genes occurring in the “Euteleostomi” strata. This category showed mainly upregulation for the hippocampus, and more downregulation for the two other types of brain cells, suggesting increased reliance on vertebrate‐specific pathways during aging for the brain. There is no genetic atavistic over‐representation; the DEGs are indeed not significantly over‐represented in the “All living organisms” stratum (Figure 4). The three types of cells showed under‐representation mostly located in the evolutionary youngest genes, from “Amniota” to “Primates” for Cerebellum cells, from “Euarchontoglires” to “Primates” strata for hippocampus cells and from “Mammalia” to “Primates” for cortex cells.

The mesenchymal cells, in contrast, showed no over‐representation nor under‐representation of DEGs in “Euteleostomi” (Figure 4). However, we note that the number of genes analyzed in mesenchymal cells is almost an order of magnitude smaller than that of the other cells. This could have reduced the statistical power to detect potential differences according to evolutionary age.

Brain aging signatures showed moderate evolutionary age shifts. It is significant for cortex and cerebellum cells, with a mean age shift of −0.74 and −0.90 categories respectively and not significant for the hippocampus with a mean age shift of −0.31 (see Table 1), while mesenchymal stem cells showed a non‐significant and minimal shift (−0.27 categories), revealing tissue‐specific manifestations of the atavism theory across different cell types.

We can conclude there is no atavistic over‐representation of the genetic changes in the most ancient genes for brain and mesenchymal stem cells suggesting a differential aging or resistance to aging for the brain tissue and stem cells, as suggested by (Nie et al. 2022; Schaum et al. 2020).

### 3.2 Atavistic Dysregulation of the Most Ancient Genes During Aging

Overall, we found an age‐dependent increase in heterogeneity in the direction of the phylogenetic position of tissues' transcriptional profiles. The number of upregulated and downregulated genes in the most ancient genes in the GenAge aging signature is almost equal, suggesting more of a reshuffling in genetic expression during aging in the most ancient genes.

The AgeMeta aging signature is dominated by downregulated genes in the “All living organisms” strata. Upregulated genes were more important in the most ancient genes in immune and ovarian cells, but not in HPC, senescent or skin cells where downregulated genes dominated in the most ancient genes. For the other types of cells, it is almost equal between up‐and downregulated genes in the most ancient genes category.

Therefore, our analyses suggest that aging involves not merely a stochastic decay but a heterogeneous cellular phylogenetic dissociation over time (see Appendix A for the significant differences using a hypergeometric test between cumulative percentage distributions of gene evolutionary ages for aging‐related gene sets compared to the baseline genome distribution).

## 4 Discussion

We tested predictions of the Atavistic Gene Expression Dissociation (AGED) hypothesis during aging using a meta‐phylostratigraphic analysis. We applied it to RNA‐seq and scRNA‐seq data from 8 different studies two meta‐analyses including different tissues: RNA‐seq during aging (Palmer et al. 2021; Tikhonov et al. 2024), scRNA‐seq data from skin cells (Zou et al. 2021), an aging meta‐analysis of the scRNA‐seq of senescent cells (Avelar et al. 2020), and brain (cortex, hippocampus and cerebellum cells) (González‐Velasco et al. 2020), immune (Lu et al. 2022), ovarian (Jin et al. 2024) and stem cells RNA‐seq (Wagner et al. 2009) (see Table 1). We found: (1) An atavistic over‐representation of differential expression in the most ancient genes and under‐representation in the evolutionary youngest genes for two multi‐tissue aging databases, and tissues covering skin, ovarian, immune, senescent and mesenchymal‐senescent cells; (2) No significant atavistic over‐representation of the differential gene expression during aging of brain cells and mesenchymal stem cells; (3) an overall age‐dependent increase of heterogeneity in the direction of the phylogenetic position of tissues' transcriptional profiles; and (4) and an overall negative evolutionary age mean shift toward the most ancient genes. Our analyses suggest that aging involves uncoordinated and tissue‐specific phylogenetic changes in gene expression, revealing a cellular dissociation in phylogenetic space during aging resembling the atavistic theory of cancer for aged cells and some tissue and loss of cellular identity (Izgi et al. 2022; Soto and Sonnenschein 2004). For the last study, they focus on the loss of cellular identity and transcriptomic changes during aging but lack the phylostratigraphic analysis we did in this work. We also found some genetic heterogeneity in the directions of the genetic changes as sometimes it is more upregulation of the most ancient genes that predominates and sometimes it is the opposite, and some cells seem not affected by these atavistic changes (brain and mesenchymal stem cells).

Numerous properties (biochemical, bioelectrical, and biomechanical) are likely affected by cells' phylogenetic status; we focused on transcriptomic analysis as expressed genes provide the only known effective method to estimate the effective phylogenetic age of a given tissue. However, it will be interesting in the future to see whether for example bioelectrical states can also be used to estimate phylogenetic position (i.e., it's not all about the genes).

To analyze the DEGs in the aging cells under various conditions through phylostratigraphic analysis, we used the evolutionary ages of 19,660 human protein‐coding genes as determined by Litman and Stein (2019). These genes were categorized into 19 major phylostrata, as outlined by Domazet‐Lošo and Tautz (2010). Phylostratigraphic analysis, while providing valuable insights into the evolutionary age of genes, is subject to several limitations that impact the accuracy and consistency of its findings. One challenge is ensuring consistency in ortholog identification across different databases (Litman and Stein 2019). Litman and Stein (2019) found a consensus across several databases but for some genes, uncertainty may remain, leading to the possibility of both false positives and negatives. Another limitation is analyzing noncoding genes. Phylostratigraphic analysis on these genes is more challenging as a significantly lower fraction of noncoding genes can be assigned a reliable evolutionary age (Litman and Stein 2019). This discrepancy may come from the rapid divergence and lineage‐specific nature of many noncoding sequences, which results in a lower likelihood of detecting homologs across distant taxa. The absence of a consistent modal value for many noncoding genes further underscores the challenge of dating their origins, necessitating reliance on statistical estimates such as the median, which may still introduce uncertainties. In this paper, we didn't analyze the evolutionary age of the noncoding genes and focused on the protein‐coding ones. The majority of noncoding genes analyzed in prior studies appear prominently from the Euteleostomi phylostratigraphic age, with the largest expansion observed during the Primata age (Litman and Stein 2019). This suggests that noncoding genes likely play critical roles in lineage‐specific regulatory innovations. Future research addressing noncoding genes would be valuable, providing deeper insights into their evolutionary trajectories and functional implications in various lineages, particularly within the “Primata” phylostratographic age, where their abundance is highest.

Senescent behavior is not restricted to proliferative arrest. Indeed, another important characteristic of senescent cells is the senescence‐associated secretory phenotype (SASP), where cells secrete inflammatory cytokines, growth factors, and proteases into their environment (Campisi 2013). From an atavistic point of view, cellular senescence may not be just a passive response to molecular damage but an active, evolutionarily conserved process that occurs when the controls of multicellularity fail and cells pursue their own goals (Khosla et al. 2020), and that plays a role in cellular interaction and adaptation to high‐stress conditions (Thomas et al. 2016). However, in the case of aging, this process is dysregulated and leads to a defect of tissue homeostasis and promotes chronic inflammation (Campisi 2013).

Heterochronic parabiosis, the surgical connection of the circulatory systems of young and old organisms, has demonstrated that aging is not irreversible but is instead highly plastic and responsive to systemic factors (Conboy et al. 2005). From the perspective of the atavistic theory of aging, the rejuvenating effects observed in heterochronic parabiosis experiments may not come from the inhibition or reduction of pro‐aging factors along with the introduction of youthful signals, but rather from the reconstitution of the multicellular integration that has been lost and the reduction of cellular phylogenetic dissociation. If aging is a trajectory of increasing cellular atavism, where cells revert to evolutionarily ancient behaviors, then exposure to young systemic factors may act as a re‐establishment of developmental/morphogenetic constraints, pulling aged cells back into the organizational structure of a more coherent, goal‐directed system.

Cancer can be understood as the failure of the computational boundary that regulates organ‐level integration in the body (Levin 2019). The cells lose multicellular integration into networks that pursue large‐scale anatomical setpoints, and function more as autonomous units that treat the rest of the body as external environment, similarly to their ancestral unicellular behaviors (Levin 2021; Bussey et al. 2017; Davies and Lineweaver 2011; Rubin 1985). Therefore, cancer is believed to occur due to failure of the regulatory networks that usually restrict individual‐level goals and favor morphogenetic goals such as bioelectric signaling, resulting in uncontrolled cellular autonomy and proliferation (Levin 2019). This reduction of the radius of cooperation/coordination among cells, from organ‐level to individual cell level, is consistent with the loss of phylogenetic age we observed across tissues with age.

In the atavistic model of cancer, cancer progression is seen as an expression of the genetic to the ancestral cellular states, which are defined by the reactivation of the genetic programs that have been inherited from the unicellular or early multicellular evolutionary stages (Lineweaver and Davies 2021; Thomas et al. 2017). While the somatic mutation theory (SMT) has been used to explain the development of cancer as a result of sequential mutations and natural selection events within the human body (Soto and Sonnenschein 2004), the atavistic model is based on the idea that ancient functionalities that were essential for early and unicellular life forms are involved in the disease. These functionalities are uncontrolled growth, lack of response to growth inhibitors and increased stress tolerance. Phylostratigraphic analysis has been used to establish that many genes associated with cancer were born during critical transitions in the evolution of life on Earth such as the rise of unicellular, eukaryotes or multicellularity (Lineweaver and Davies 2021).

Also, in the active inference framework (Friston 2012; Kirchhoff et al. 2018; Parr et al. 2022), cells are described as having a model of themselves and the outside world (Kuchling et al. 2020; Pio‐Lopez et al. 2022). Maybe evolutionary stage is part of their self‐model, and the transcriptional changes we see are a readout of the change of this model with age.

Atavistic dysregulation during aging suggests new avenues for longevity therapeutics: strategies should focus on restoring and reinforcing multicellular coherence, re‐establishing a multi‐cellular and evolutionary younger genetic expression that preserves tissue and organ integration (perhaps by stimuli that remind cells of their modern evolutionary status). In the context of aging as a loss of morphostatic information and goal‐directedness (Pio‐Lopez and Levin 2024; Pio‐Lopez et al. 2024), cellular dissociation in morphogenetic space suggests that giving evolutionarily recent information for the organism may counteract aging. We suggest this is the beginning of a roadmap that pursues healthspan and longevity by managing the processing, by cells and tissues, of information across a wide range of temporal and spatial scales.

## Author Contributions

Conceptualized study: L.P.‐L., M.L.; performed bioinformatics analysis: L.P.‐L; interpreted data: L.P.‐L., M.L.; wrote and edited manuscript: L.P.‐L., M.L.

## Funding

This work was supported by Templeton World Charity Foundation (TWCF0606) and Astonishing Labs.

## Conflicts of Interest

The Levin lab has a sponsored research agreement with Astonishing Labs, a company seeking to impact the longevity and biomedicine of the aging field.

## Data Availability

The code for data analysis is available on Github at this address: https://github.com/LPioL/atavisticDissociation.

## Appendix A

Cumulative percentage distributions of gene evolutionary ages for aging‐related gene sets (blue lines) compared to the baseline genome distribution (orange lines). Red stars indicate significant over‐representation and black stars indicate significant under‐representation at each evolutionary age cutoff (FDR‐corrected *p* < 0.05, cumulative hypergeometric test).

## References

[acel70305-bib-0001] Ashburner, M. , C. A. Ball , J. A. Blake , et al. 2000. “Gene Ontology: Tool for the Unification of Biology. The Gene Ontology Consortium.” Nature Genetics 25, no. 1: 25–29. 10802651 10.1038/75556 PMC3037419

[acel70305-bib-0002] Austad, S. N. , and J. M. Hoffman . 2018. “Is Antagonistic Pleiotropy Ubiquitous in Aging Biology?” Evolution, Medicine, and Public Health 2018, no. 1: 287–294. 30524730 10.1093/emph/eoy033 PMC6276058

[acel70305-bib-0003] Avelar, R. A. , J. G. Ortega , R. Tacutu , et al. 2020. “A Multidimensional Systems Biology Analysis of Cellular Senescence in Aging and Disease.” Genome Biology 21, no. 1: 91. 32264951 10.1186/s13059-020-01990-9 PMC7333371

[acel70305-bib-0004] Bussey, K. J. , L. H. Cisneros , C. H. Lineweaver , and P. C. W. Davies . 2017. “Ancestral Gene Regulatory Networks Drive Cancer.” Proceedings of the National Academy of Sciences of the United States of America 114, no. 24: 6160–6162. 28584134 10.1073/pnas.1706990114 PMC5474827

[acel70305-bib-0005] Campisi, J. 2013. “Aging, Cellular Senescence, and Cancer.” Annual Review of Physiology 75, no. 1: 685–705. 10.1146/annurev-physiol-030212-183653 PMC4166529 23140366

[acel70305-bib-0006] Chernet, B. T. , and M. Levin . 2013. “Endogenous Voltage Potentials and the Microenvironment: Bioelectric Signals That Reveal, Induce and Normalize Cancer.” Journal of Clinical & Experimental Oncology Suppl 1: S1‐002. 10.4172/2324-9110.S1-002 PMC4267524 25525610

[acel70305-bib-0007] Conboy, I. M. , M. J. Conboy , A. J. Wagers , E. R. Girma , I. L. Weissman , and T. A. Rando . 2005. “Rejuvenation of Aged Progenitor Cells by Exposure to a Young Systemic Environment.” Nature 433, no. 7027: 760–764. 15716955 10.1038/nature03260

[acel70305-bib-0008] Davies, P. C. , and C. H. Lineweaver . 2011. “Cancer Tumors as Metazoa 1.0: Tapping Genes of Ancient Ancestors.” Physiological Biology 8, no. 1: 015001. 10.1088/1478-3975/8/1/015001 PMC3148211 21301065

[acel70305-bib-0009] de Magalhães, J. P. 2011. “The Biology of Ageing: A Primer.” In An Introduction to Gerontology, edited by I. Stuart‐Hamilton , 21–47. Cambridge University Press.

[acel70305-bib-0010] de Magalhães, J. P. 2023. “Ageing as a Software Design Flaw.” Genome Biology 24, no. 1: 51. 36973715 10.1186/s13059-023-02888-y PMC10042583

[acel70305-bib-0011] de Magalhães, J. P. , and G. M. Church . 2005. “Genomes Optimize Reproduction: Aging as a Consequence of the Developmental Program.” Physiology (Bethesda) 20, no. 4: 252–259. 16024513 10.1152/physiol.00010.2005

[acel70305-bib-0012] Domazet‐Lošo, T. , J. Brajković , and D. Tautz . 2007. “A Phylostratigraphy Approach to Uncover the Genomic History of Major Adaptations in Metazoan Lineages.” Trends in Genetics 23, no. 11: 533–539. 18029048 10.1016/j.tig.2007.08.014

[acel70305-bib-0013] Domazet‐Lošo, T. , and D. Tautz . 2010. “Phylostratigraphic Tracking of Cancer Genes Suggests a Link to the Emergence of Multicellularity in Metazoa.” BMC Biology 8: 66. 20492640 10.1186/1741-7007-8-66 PMC2880965

[acel70305-bib-0014] Duran‐Nebreda, S. , A. Bonforti , R. Montañez , S. Valverde , and R. Solé . 2016. “Emergence of Proto‐Organisms From Bistable Stochastic Differentiation and Adhesion.” Journal of the Royal Society Interface 13, no. 117: 20160108. 27053655 10.1098/rsif.2016.0108 PMC4874432

[acel70305-bib-0015] Fields, C. , and M. Levin . 2022. “Competency in Navigating Arbitrary Spaces as an Invariant for Analyzing Cognition in Diverse Embodiments.” Entropy (Basel) 24, no. 6: 819. 35741540 10.3390/e24060819 PMC9222757

[acel70305-bib-0027] Friston, K. 2012. “A Free Energy Principle for Biological Systems.” Entropy (Basel) 14, no. 11: 2100–2121. 23204829 10.3390/e14112100 PMC3510653

[acel70305-bib-0016] Friston, K. , M. Levin , B. Sengupta , and G. Pezzulo . 2015. “Knowing One's Place: A Free‐Energy Approach to Pattern Regulation.” Journal of the Royal Society Interface 12, no. 105: 20141383. 25788538 10.1098/rsif.2014.1383 PMC4387527

[acel70305-bib-0017] Gems, D. 2022. “The Hyperfunction Theory: An Emerging Paradigm for the Biology of Aging.” Ageing Research Reviews 74: 101557. 34990845 10.1016/j.arr.2021.101557 PMC7612201

[acel70305-bib-0018] Gilbert, S. F. , J. M. Opitz , and R. A. Raff . 1996. “Resynthesizing Evolutionary and Developmental Biology.” Developmental Biology 173, no. 2: 357–372. 8605997 10.1006/dbio.1996.0032

[acel70305-bib-0019] Gladyshev, V. N. , S. B. Kritchevsky , S. G. Clarke , et al. 2021. “Molecular Damage in Aging.” Nature Aging 1, no. 12: 1096–1106. 36846190 10.1038/s43587-021-00150-3 PMC9957516

[acel70305-bib-0020] González‐Velasco, O. , D. Papy‐García , G. le Douaron , J. M. Sánchez‐Santos , and J. de Las Rivas . 2020. “Transcriptomic Landscape, Gene Signatures and Regulatory Profile of Aging in the Human Brain.” Biochimica et Biophysica Acta (BBA)—Gene Regulatory Mechanisms 1863, no. 6: 194491. 32006715 10.1016/j.bbagrm.2020.194491

[acel70305-bib-0021] Harris, A. K. 2018. “The Need for a Concept of Shape Homeostasis.” Biosystems 173: 65–72. 30268925 10.1016/j.biosystems.2018.09.012

[acel70305-bib-0022] Hayflick, L. 2007. “Biological Aging Is No Longer an Unsolved Problem.” Annals of the New York Academy of Sciences 1100, no. 1: 1–13. 17460161 10.1196/annals.1395.001

[acel70305-bib-0023] Izgi, H. , D. Han , U. Isildak , et al. 2022. “Inter‐Tissue Convergence of Gene Expression During Ageing Suggests Age‐Related Loss of Tissue and Cellular Identity.” eLife 11: e68048. 35098922 10.7554/eLife.68048 PMC8880995

[acel70305-bib-0024] Jeffery, W. R. , and R. A. Raff . 1983. “Time, Space, and Pattern in Embryonic Development.” In MBL Lectures in Biology, vol. 2, xvii–395. A.R. Liss.

[acel70305-bib-0025] Jin, C. , X. Wang , J. Yang , et al. 2024. “Molecular and Genetic Insights Into Human Ovarian Aging From Single‐Nuclei Multi‐Omics Analyses.” Nature Aging 5: 1–290. 10.1038/s43587-024-00762-5 PMC11839473 39578560

[acel70305-bib-0026] Kanehisa, M. , M. Furumichi , M. Tanabe , Y. Sato , and K. Morishima . 2017. “KEGG: New Perspectives on Genomes, Pathways, Diseases and Drugs.” Nucleic Acids Research 45, no. D1: D353–D361. 27899662 10.1093/nar/gkw1092 PMC5210567

[acel70305-bib-0028] Khosla, S. , J. N. Farr , T. Tchkonia , and J. L. Kirkland . 2020. “The Role of Cellular Senescence in Ageing and Endocrine Disease.” Nature Reviews. Endocrinology 16, no. 5: 263–275. 10.1038/s41574-020-0335-y PMC7227781 32161396

[acel70305-bib-0029] Kirchhoff, M. , T. Parr , E. Palacios , K. Friston , and J. Kiverstein . 2018. “The Markov Blankets of Life: Autonomy, Active Inference and the Free Energy Principle.” Journal of the Royal Society Interface 15, no. 138: 20170792. 29343629 10.1098/rsif.2017.0792 PMC5805980

[acel70305-bib-0030] Kirkwood, T. B. , and S. Melov . 2011. “On the Programmed/Non‐Programmed Nature of Ageing Within the Life History.” Current Biology 21, no. 18: R701–R707. 21959160 10.1016/j.cub.2011.07.020

[acel70305-bib-0031] Kuchling, F. , K. Friston , G. Georgiev , and M. Levin . 2020. “Morphogenesis as Bayesian Inference: A Variational Approach to Pattern Formation and Control in Complex Biological Systems.” Physics of Life Reviews 33: 88–108. 31320316 10.1016/j.plrev.2019.06.001

[acel70305-bib-0032] Lagasse, E. , and M. Levin . 2023. “Future Medicine: From Molecular Pathways to the Collective Intelligence of the Body.” Trends in Molecular Medicine 29: 687–710. 37481382 10.1016/j.molmed.2023.06.007 PMC10527237

[acel70305-bib-0033] Levin, M. 2019. “The Computational Boundary of a “Self”: Developmental Bioelectricity Drives Multicellularity and Scale‐Free Cognition.” Frontiers in Psychology 10, no. 2688: 2688. 31920779 10.3389/fpsyg.2019.02688 PMC6923654

[acel70305-bib-0034] Levin, M. 2021. “Bioelectrical Approaches to Cancer as a Problem of the Scaling of the Cellular Self.” Progress in Biophysics and Molecular Biology 165: 102–113. 33961843 10.1016/j.pbiomolbio.2021.04.007

[acel70305-bib-0035] Levin, M. 2023a. “Collective Intelligence of Morphogenesis as a Teleonomic Process.” In Evolution ‘on Purpose’: Teleonomy in Living Systems, edited by P. A. Corning , S. A. Kauffman , D. Noble , et al., 175–198. MIT Press.

[acel70305-bib-0036] Levin, M. 2023b. “Bioelectric Networks: The Cognitive Glue Enabling Evolutionary Scaling From Physiology to Mind.” Animal Cognition 26: 1865–1891. 37204591 10.1007/s10071-023-01780-3 PMC10770221

[acel70305-bib-0037] Levin, M. 2024. “The Multiscale Wisdom of the Body: Collective Intelligence as a Tractable Interface for Next‐Generation Biomedicine.” BioEssays 47: e202400196. 39623868 10.1002/bies.202400196 PMC11848127

[acel70305-bib-0038] Lidsky, P. V. , and R. Andino . 2020. “Epidemics as an Adaptive Driving Force Determining Lifespan Setpoints.” Proceedings of the National Academy of Sciences of the United States of America 117, no. 30: 17937–17948. 32651271 10.1073/pnas.1920988117 PMC7395509

[acel70305-bib-0039] Lidsky, P. V. , and R. Andino . 2022. “Could Aging Evolve as a Pathogen Control Strategy?” Trends in Ecology & Evolution 37, no. 12: 1046–1057. 36096982 10.1016/j.tree.2022.08.003

[acel70305-bib-0040] Lidsky, P. V. , J. Yuan , and R. Andino . 2023. “Reconsidering Life History Theory Amid Infectious Diseases.” Trends in Ecology & Evolution 38, no. 8: 699–700. 37236881 10.1016/j.tree.2023.05.004

[acel70305-bib-0041] Lidsky, P. V. , J. Yuan , J. M. Rulison , and R. Andino‐Pavlovsky . 2022. “Is Aging an Inevitable Characteristic of Organic Life or an Evolutionary Adaptation?” Biochemistry (Moscow) 87, no. 12: 1413–1445. 36717438 10.1134/S0006297922120021 PMC9839256

[acel70305-bib-0042] Lineweaver, C. H. , and P. C. W. Davies . 2021. “Comparison of the Atavistic Model of Cancer to Somatic Mutation Theory: Phylostratigraphic Analyses Support the Atavistic Model.” In The Physics of Cancer: Research Advances, 243–261. World Scientific.

[acel70305-bib-0043] Litman, T. , and W. D. Stein . 2019. “Obtaining Estimates for the Ages of All the Protein‐Coding Genes and Most of the Ontology‐Identified Noncoding Genes of the Human Genome, Assigned to 19 Phylostrata.” Seminars in Oncology 46: 3–9. 30558821 10.1053/j.seminoncol.2018.11.002

[acel70305-bib-0044] López‐Otín, C. , M. A. Blasco , L. Partridge , M. Serrano , and G. Kroemer . 2023. “Hallmarks of Aging: An Expanding Universe.” Cell 186, no. 2: 243–278. 36599349 10.1016/j.cell.2022.11.001

[acel70305-bib-0045] Lu, J. , R. Ahmad , T. Nguyen , et al. 2022. “Heterogeneity and Transcriptome Changes of Human CD8(+) T Cells Across Nine Decades of Life.” Nature Communications 13, no. 1: 5128. 10.1038/s41467-022-32869-x PMC9436929 36050300

[acel70305-bib-0046] McGhee, G. R. 2007. The Geometry of Evolution: Adaptive Landscapes and Theoretical Morphospaces, xii–200. Cambridge University Press.

[acel70305-bib-0047] McMillen, P. , and M. Levin . 2024. “Collective Intelligence: A Unifying Concept for Integrating Biology Across Scales and Substrates.” Communications Biology 7, no. 1: 378. 38548821 10.1038/s42003-024-06037-4 PMC10978875

[acel70305-bib-0048] Moore, D. , S. I. Walker , and M. Levin . 2017. “Cancer as a Disorder of Patterning Information: Computational and Biophysical Perspectives on the Cancer Problem.” Convergent Science Physical Oncology 3: 043001.

[acel70305-bib-0049] Newman, S. A. , and R. Bhat . 2009. “Dynamical Patterning Modules: A ‘Pattern Language’ for Development and Evolution of Multicellular Form.” International Journal of Developmental Biology 53, no. 5–6: 693–705. 19378259 10.1387/ijdb.072481sn

[acel70305-bib-0050] Nie, C. , Y. Li , R. Li , et al. 2022. “Distinct Biological Ages of Organs and Systems Identified From a Multi‐Omics Study.” Cell Reports 38, no. 10: 110459. 35263580 10.1016/j.celrep.2022.110459

[acel70305-bib-0051] Nijenhuis, E. R. , P. Spinhoven , R. van Dyck , O. van der Hart , and J. Vanderlinden . 1998. “Degree of Somatoform and Psychological Dissociation in Dissociative Disorder Is Correlated With Reported Trauma.” Journal of Traumatic Stress 11, no. 4: 711–730. 9870223 10.1023/A:1024493332751

[acel70305-bib-0052] Ollé‐Vila, A. , S. Duran‐Nebreda , N. Conde‐Pueyo , R. Montañez , and R. Solé . 2016. “A Morphospace for Synthetic Organs and Organoids: The Possible and the Actual.” Integrative Biology 8, no. 4: 485–503. 27032985 10.1039/c5ib00324e

[acel70305-bib-0053] Palmer, D. , F. Fabris , A. Doherty , A. A. Freitas , and J. P. de Magalhães . 2021. “Ageing Transcriptome Meta‐Analysis Reveals Similarities and Differences Between Key Mammalian Tissues.” Aging (Albany NY) 13, no. 3: 3313–3341. 33611312 10.18632/aging.202648 PMC7906136

[acel70305-bib-0054] Parr, T. , G. Pezzulo , and K. J. Friston . 2022. Active Inference: The Free Energy Principle in Mind, Brain, and Behavior. MIT Press.

[acel70305-bib-0055] Partridge, L. , J. Deelen , and P. E. Slagboom . 2018. “Facing up to the Global Challenges of Ageing.” Nature 561, no. 7721: 45–56. 30185958 10.1038/s41586-018-0457-8

[acel70305-bib-0056] Pezzulo, G. , and M. Levin . 2015. “Re‐Membering the Body: Applications of Computational Neuroscience to the Top‐Down Control of Regeneration of Limbs and Other Complex Organs.” Integrative Biology 7, no. 12: 1487–1517. 26571046 10.1039/c5ib00221d PMC4667987

[acel70305-bib-0057] Pezzulo, G. , and M. Levin . 2016. “Top‐Down Models in Biology: Explanation and Control of Complex Living Systems Above the Molecular Level.” Journal of the Royal Society Interface 13, no. 124: 20160555. 27807271 10.1098/rsif.2016.0555 PMC5134011

[acel70305-bib-0058] Pio‐Lopez, L. , B. Hartl , and M. Levin . 2025. “Aging as a Loss of Goal‐Directedness: An Evolutionary Simulation and Analysis Unifying Regeneration with Anatomical Rejuvenation.” Advanced Science: e09872. 10.1002/advs.202509872. 41082360 PMC12697880

[acel70305-bib-0059] Pio‐Lopez, L. , F. Kuchling , A. Tung , G. Pezzulo , and M. Levin . 2022. “Active Inference, Morphogenesis, and Computational Psychiatry.” Frontiers in Computational Neuroscience 16: 988977. 36507307 10.3389/fncom.2022.988977 PMC9731232

[acel70305-bib-0060] Pio‐Lopez, L. , and M. Levin . 2024. “Aging as a Loss of Morphostatic Information: A Developmental Bioelectricity Perspective.” Ageing Research Reviews 97: 102310. 38636560 10.1016/j.arr.2024.102310

[acel70305-bib-0061] Raff, R. A. 1996. The Shape of Life: Genes, Development, and the Evolution of Animal Form, xxiii–520. University of Chicago Press.

[acel70305-bib-0062] Rasskin‐Gutman, D. , and J. C. Izpisua‐Belmonte . 2004. “Theoretical Morphology of Developmental Asymmetries.” BioEssays 26, no. 4: 405–412. 15057938 10.1002/bies.10410

[acel70305-bib-0063] Rubin, H. 1985. “Cancer as a Dynamic Developmental Disorder.” Cancer Research 45, no. 7: 2935–2942. 3891078

[acel70305-bib-0064] Rubin, H. 2006. “What Keeps Cells in Tissues Behaving Normally in the Face of Myriad Mutations?” BioEssays 28, no. 5: 515–524. 16615084 10.1002/bies.20403

[acel70305-bib-0065] Rubin, H. 2007. “Ordered Heterogeneity and Its Decline in Cancer and Aging.” Advances in Cancer Research 98: 117–147. 17433909 10.1016/S0065-230X(06)98004-X

[acel70305-bib-0066] Rubin, H. , M. Chow , and A. Yao . 1996. “Cellular Aging, Destabilization, and Cancer.” Proceedings of the National Academy of Sciences of the United States of America 93, no. 5: 1825–1830. 8700843 10.1073/pnas.93.5.1825 PMC39866

[acel70305-bib-0067] Schaum, N. , B. Lehallier , O. Hahn , et al. 2020. “Ageing Hallmarks Exhibit Organ‐Specific Temporal Signatures.” Nature 583, no. 7817: 596–602. 32669715 10.1038/s41586-020-2499-y PMC7757734

[acel70305-bib-0068] Skulachev, M. V. , and V. P. Skulachev . 2014. “New Data on Programmed Aging—Slow Phenoptosis.” Biochemistry (Moscow) 79: 977–993. 25519058 10.1134/S0006297914100010

[acel70305-bib-0069] Soto, A. M. , and C. Sonnenschein . 2004. “The Somatic Mutation Theory of Cancer: Growing Problems With the Paradigm?” BioEssays 26, no. 10: 1097–1107. 15382143 10.1002/bies.20087

[acel70305-bib-0070] Stone, J. R. 1997. “The Spirit of D'arcy Thompson Dwells in Empirical Morphospace.” Mathematical Biosciences 142, no. 1: 13–30. 9125858 10.1016/s0025-5564(96)00186-1

[acel70305-bib-0071] Subramanian, A. , P. Tamayo , V. K. Mootha , et al. 2005. “Gene Set Enrichment Analysis: A Knowledge‐Based Approach for Interpreting Genome‐Wide Expression Profiles.” Proceedings of the National Academy of Sciences of the United States of America 102, no. 43: 15545–15550. 16199517 10.1073/pnas.0506580102 PMC1239896

[acel70305-bib-0072] Thomas, F. , R. M. Nesse , R. Gatenby , et al. 2016. “Evolutionary Ecology of Organs: A Missing Link in Cancer Development?” Trends Cancer 2, no. 8: 409–415. 28741494 10.1016/j.trecan.2016.06.009

[acel70305-bib-0073] Thomas, F. , B. Ujvari , F. Renaud , and M. Vincent . 2017. “Cancer Adaptations: Atavism, de Novo Selection, or Something in Between?” BioEssays 39, no. 8: 1700039. 10.1002/bies.201700039 28691339

[acel70305-bib-0074] Tikhonov, S. , M. Batin , V. N. Gladyshev , S. E. Dmitriev , and A. Tyshkovskiy . 2024. “AgeMeta: Quantitative Gene Expression Database of Mammalian Aging.” Biochemistry (Mosc) 89, no. 2: 313–321. 38622098 10.1134/S000629792402010X

[acel70305-bib-0075] Wagner, W. , S. Bork , P. Horn , et al. 2009. “Aging and Replicative Senescence Have Related Effects on Human Stem and Progenitor Cells.” PLoS One 4, no. 6: e5846. 19513108 10.1371/journal.pone.0005846 PMC2688074

[acel70305-bib-0076] Waller, G. , K. Hamilton , P. Elliott , et al. 2001. “Somatoform Dissociation, Psychological Dissociation, and Specific Forms of Trauma.” Journal of Trauma & Dissociation 1, no. 4: 81–98.

[acel70305-bib-0077] Zou, Z. , X. Long , Q. Zhao , et al. 2021. “A Single‐Cell Transcriptomic Atlas of Human Skin Aging.” Developmental Cell 56, no. 3: 383–397.e8. 33238152 10.1016/j.devcel.2020.11.002

